# Co-Existence of Endometriosis with Ovarian Dermoid Cysts: A Retrospective Cohort Study

**DOI:** 10.3390/jcm12196308

**Published:** 2023-09-30

**Authors:** Dimitrios Rafail Kalaitzopoulos, Nicolas Samartzis, Markus Eberhard, Georgios Grigoriadis, Dimosthenis Miliaras, Alexis Papanikolaou, Angelos Daniilidis

**Affiliations:** 1 Department of Obstetrics and Gynecology, Cantonal Hospital of Schaffhausen, 8208 Schaffhausen, Switzerland; nicolas.samartzis@spitaeler-sh.ch (N.S.); markus.eberhard@spitaeler-sh.ch (M.E.); 2 2nd University Department in Obstetrics and Gynecology, Hippokratio General Hospital, School of Medicine, Aristotle University of Thessaloniki, 54643 Thessaloniki, Greece; drgeorgiosgrigoriadis@gmail.com (G.G.);; 3 Laboratory of Histology and Embryology, School of Medicine, Aristotle University of Thessaloniki, 54643 Thessaloniki, Greece; miliaras@auth.gr; 4 1st University Department in Obstetrics and Gynaecology, Papageorgiou General Hospital, School of Medicine, Aristotle University of Thessaloniki, 54643 Thessaloniki, Greece

**Keywords:** endometriosis, ovarian dermoid cysts, teratoma, co-existence, fertility, diagnosis

## Abstract

Both endometriosis and ovarian dermoid cysts are benign conditions characterized by the presence of well-differentiated tissues in ectopic locations. The presence and surgical excision of these entities can potentially impact ovarian reserves, contributing to reduced chances of future pregnancy. The objective of our study is to investigate the bidirectional association between endometriosis and ovarian dermoid cysts, as well as to analyze the clinical characteristics of patients diagnosed with both conditions. A retrospective cohort study was conducted, including women who underwent laparoscopy and received histological diagnoses of endometriosis and/or dermoid cysts between 2011 and 2019 at the Cantonal Hospital of Schaffhausen. We identified 985 women with endometriosis and 83 women with ovarian dermoid cysts. Among these groups, 22 women presented with both endometriosis and ovarian dermoid cysts. The majority of the above patients had endometriosis stage rASRM I-II (72.7%), with peritoneal endometriosis being the most common phenotype of endometriosis (77.2%). Out of the 14 patients with a desire for future pregnancy, the majority (11/14, 78.5%) had an EFI score of 7–8. The prevalence of bilateral ovarian dermoid cysts was higher in women with both ovarian dermoid cysts and endometriosis in comparison to women with ovarian dermoid cysts without endometriosis (18% vs. 6.5%). Our study revealed that 26.5% of women with ovarian dermoid cysts also had endometriosis, a notably higher prevalence than observed in the general population. Clinicians should be aware of this co-existence, and preoperative counseling should be an integral part of the care plan for affected individuals, where the potential risks and the available options for fertility preservation should be discussed in detail.

## 1. Introduction

Endometriosis is a benign estrogen-dependent chronic inflammatory disease with a prevalence of about 10% in women of reproductive age [1]. Several theories have been proposed for the pathogenesis of endometriosis [1]. The most widely accepted theory is the theory of retrograde menstruation, which was first described by Sampson; however, among the other theories, there is also the theory of undifferentiated stem cells, which have the ability to differentiate into one or more cell types [1]. The complex nature of this entity could be reflected on the lack of consensus among different societies regarding a common classification system and discrepancies in the proposed treatment strategies [2,3,4].

Patients with endometriosis experience a wide range of symptoms; however, the two major clinical manifestations of this condition are pain (including chronic pelvic pain, dysmenorrhea, dysuria, dyschezia, and dyspareunia) and infertility [1]. The symptoms mentioned above progressively impact various aspects of a woman’s daily life, including sleep, sexual activity, social relationships, work ability, and financial status [5]. These impairments significantly affect the quality of life of endometriosis patients. In terms of the research on the quality of life in women with endometriosis, there has been a growing focus in recent years. Several questionnaires for assessing quality of life have been developed, translated, and validated [5,6,7]. The most commonly used ones include the Short Form 36 (SF-36), the Short Form 12 (SF-12), and the World Health Organization Quality of Life Assessment-BREF (WHOQOL-BREF) [8]. Endometriosis is related to high direct healthcare costs, such as physician visits, surgery, costs of medications, and diagnostic procedures, and the indirect costs of productivity loss (absence from work, reduced productivity at work, additional support with household activities), comparable to those of major chronic diseases such as diabetes, Crohn’s disease, and rheumatoid arthritis [9]. Approximately 50% of women with infertility suffer from endometriosis, and about 6-9% of women who undergo assisted reproductive technology (ART) treatment are diagnosed with endometriosis [10]. The pathophysiology of how endometriosis contributes to infertility is not completely elucidated. The proposed mechanisms for endometriosis-associated infertility include dyspareunia, inflammation, anatomical changes through adhesions, impaired ovarian function, and reduced endometrial receptivity [11,12]. Additionally, endometriosis surgery poses a risk of an iatrogenic reduction of the ovarian reserve [11].

Ovarian dermoid cysts, also known as cystic teratomas, are benign germinal tissue tumors that arrive from the totipotent cells of the ectoderm, mesoderm, and endoderm with a prevalence of 10 to 25% in women of reproductive age [13]. Although ovarian dermoid cysts are usually asymptomatic, due to the lack of clear recommendations, many practitioners tend to excise them to prevent ovarian torsion and malignant transformations. The treatment of dermoid cysts could impact future fertility, as excision surgery may reduce the ovarian reserve and lead to pelvic adhesions [14].

Both endometriosis and dermoid ovarian cysts are benign conditions characterized by the presence of well-differentiated tissues in ectopic locations. As previously mentioned, a similar pathophysiological theory involving undifferentiated cells that can differentiate into one or more cell types has been proposed for both endometriosis and ovarian dermoid cysts [15].

The coexistence of both entities has been poorly examined. A previous study found that ovarian dermoid cysts, constituting 1.2%, are the second most common ovarian finding in women with endometriosis, after serous cysts [16]. Another study, which examined the prevalence of endometriosis in women with ovarian dermoid cysts, reported a prevalence of 22.5% [17]. To the best of our knowledge, there is no available study that has examined the coexistence of these two entities bidirectionally.

The aim of our study is to examine the association between endometriosis and ovarian dermoid cysts bidirectionally and to analyze the clinical characteristics of patients with both entities.

## 2. Methods

A retrospective cohort study was conducted, including patients who underwent laparoscopic surgery between 2011 and 2019 at a certified endometriosis clinic in the Cantonal Hospital of Schaffhausen. The inclusion criteria were (i) premenopausal women who underwent a laparoscopic surgery between 2011 and 2019 in the Cantonal Hospital of Schaffhausen and (ii) were histologically diagnosed with endometriosis and/or an ovarian dermoid cyst either during a laparoscopy between 2011 and 2019 or based on a histological report obtained before this time period. The exclusion criteria were (i) postmenopausal patients and (ii) women with an absence of histologically verified endometriosis or dermoid cyst.

All the patients included in this study had preoperative counseling, including a detailed medical history and accurate physical and imaging examinations (via ultrasound and, in some cases, magnetic resonance imaging (MRI)). Each laparoscopy was performed by a team, including at least one experienced endoscopic surgeon (Board-Certified Gynecological Surgeon by Swiss Society of Obstetrics and Gynecology), using a 30° camera optic and maintaining a CO_2_-pneumoperitoneum between 10 and 14 mmHg under general anesthesia. All procedures were recorded and saved in video format. The abdominal cavity was systematically inspected from the upper abdomen to the pelvis, and a biopsy was performed whenever a suspected endometriosis lesion was identified. Biopsies were sent for histopathological examination to the Institute of Pathology, the Cantonal Hospital of Winterthur. The revised American Society of Reproductive Medicine (rASRM) and #ENZIAN were assessed according to the intraoperative findings. Additionally, the Endometriosis Fertility Index (EFI) was calculated for women with a desire of future pregnancy.

The rASRM classification is the most widely used endometriosis classification worldwide and describes the severity of endometriosis and divides patients into four stages (minimal (stage I), mild (stage II), moderate (stage III), and severe (stage IV) based on a summary of points corresponding to the size and localization of endometriosis lesions [18]. The #ENZIAN classification, published in 2021, is the revised version of the #ENZIAN classification, which combines information about the morphology, localization, and severity of endometriosis [19]. The #ENZIAN classification is more complicated to use than the rASRM classification, However, it can be used preoperatively in cases of suspected endometriosis. The EFI score is a 10-point scoring system for women with endometriosis and a desire for a future pregnancy that assesses, with a good predictive value (AUC 0.71, 95% CI 0.65–0.80), the possibility of natural conception after surgery [20,21].

The primary outcomes of our study were the prevalence of ovarian dermoid cysts in women with endometriosis and the prevalence of endometriosis in women with ovarian dermoid cysts. The secondary outcomes were the clinical characteristics (such as laterality and size of ovarian dermoid cysts, rASRM endometriosis stage, and #ENZIAN and EFI score) of patients with the coexistence of the above entities.

All the records of patients who underwent laparoscopic surgery during this period were reviewed, and the data of patients with endometriosis and/or ovarian dermoid cysts were extracted into excel forms.

Baseline characteristics such as age, BMI, indication for surgery, size, laterality and side of the ovarian dermoid cysts, and stage of endometriosis. according to rASRM, #ENZIAN, and if applicable, the EFI scores, were analyzed.

The study protocol was accepted by the responsible cantonal Ethics Committee of Zurich (2020-02718). This study is reported following the Strengthening the Reporting of Observational Studies in Epidemiology (STROBE) statement [22].

The statistical analysis was performed using SPSS (version 27.0, IBM). The mean and standard deviation were calculated for continuous variables, while percentages were calculated for categorical variables. Chi-square was used for the comparison between categorical variables, and the *t*-test was used for continuous variables. A *p*-value < 0.05 was considered statistically significant.

## 3. Results

**Figure 1:**
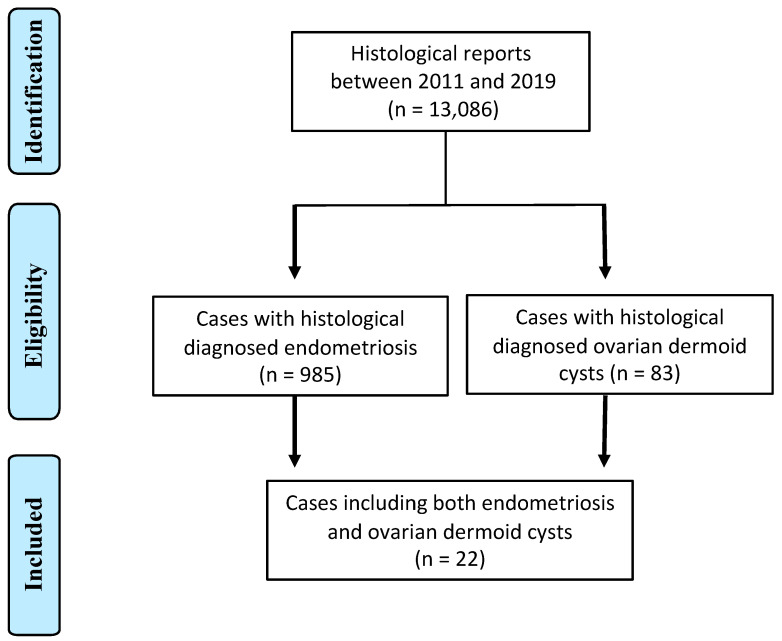
PRISMA Flow Diagram.

After reviewing the medical records, 985 women with endometriosis and 83 women with ovarian dermoid cysts were identified. Among the above groups, 22 women presented with both endometriosis and ovarian dermoid cysts (Figure 1).

The mean age of the women with the coexistence of endometriosis and ovarian dermoid cyst was 31.2 (SD 8.8) years, while the mean BMI was 22.9 (4.45) kg/m^2^. Eight patients underwent hormonal treatment preoperatively (8/22, 36.3%). The most common endometriosis-associated symptoms in this group were dysmenorrhea (11/22, 50%) and dyspareunia (6/22, 27.2%) (Table 1).

**Figure 2:**
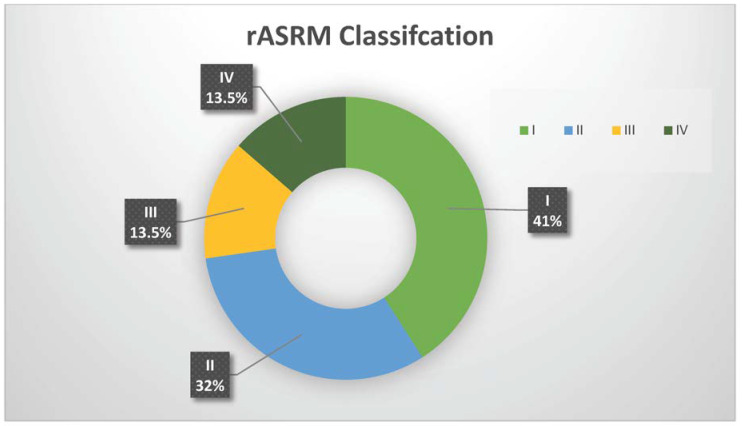
Endometriosis rASRM stage in women with co-existence of endometriosis and ovarian dermoid cysts.

In the above group, most women had endometriosis stage rASRM I-II (16/22, 72.7%), while the remaining had stage rASRM III-IV (6/22, 27.3%) (Figure 2). According to the #ENZIAN classification, the most common phenotype of endometriosis in women with both endometriosis and ovarian dermoid cysts was peritoneal endometriosis (nine women with P1 and eight women with P2). Two patients had ovarian endometrioma, while two other patients had deep endometriosis in the rectovaginal septum and three in the rectum. Three of the included patients were diagnosed with adenomyosis (Table 2).

Most of patients with both an ovarian dermoid cyst and endometriosis did not have children (15/22, 68%), while 14 patients had the desire for a future pregnancy. The vast majority of this group (11/14, 78.5%) had an EFI score of 7–8 (Table 3).

**Figure 3:**
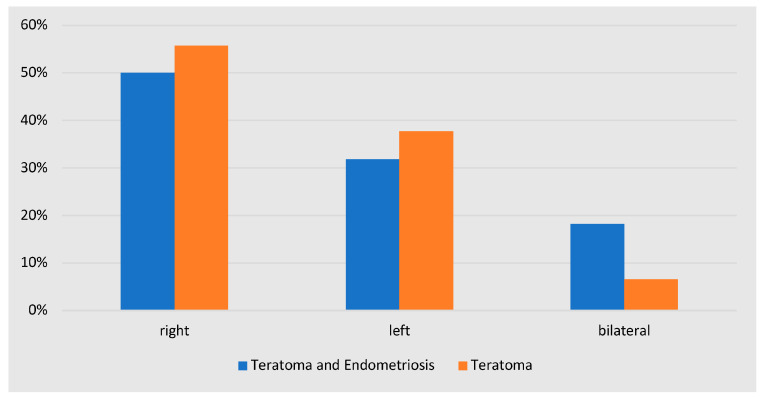
Localization of dermoid cysts in women with and without co-existence of endometriosis.

None of the included patients were diagnosed with malignant ovarian dermoid cysts. Regarding the laterality of ovarian dermoid cysts, the prevalence of bilateral ovarian dermoid cysts was higher in women with both ovarian dermoid cysts and endometriosis in comparison to women with ovarian dermoid cysts without endometriosis (18% vs. 6.5%); however this difference was not statistically significant (*p* = 0.159). The side (right/left) of the teratomas was also similar between the aforementioned groups (*p* = 0.283) (Figure 3).

Most of the patients in our population with ovarian dermoid cysts and endometriosis underwent a cystectomy for the ovarian dermoid cyst (20/22, 90.9%). Only two patients with ovarian cysts, measuring 10 cm and 16 cm, respectively, underwent an oophorectomy.

## 4. Discussion

Our study demonstrated a prevalence of endometriosis in women with dermoid cysts of 26.5% (22/83). On the other hand, the prevalence of dermoid cysts in women with endometriosis was 2.2%. To the best of our knowledge, this is the first study to examine the bidirectional association between these two entities. The observed prevalences for both associations were consistent with previous studies [16,17].

The prevalence of endometriosis in women with dermoid cysts is much higher compared to that of the general population of reproductive-age women, which is about 10% [1]. With regard to endometriosis-associated pain, dysmenorrhea was the most common symptom, with a 50% prevalence. Regarding the severity of endometriosis in women with ovarian dermoid cysts and endometriosis, the majority of the patients had peritoneal endometriosis (77.2%, 17/22) at a rASRM I-II stage (72.7%, 16/22). Ovarian endometriomas were found in two cases of the above group.

The mean dermoid cyst size in the women with both endometriosis and dermoid ovarian cysts was about 5cm. In addition, these women had a higher prevalence of bilateral dermoid cysts in comparison to the women with only dermoid cysts (18% vs. 6.5%). The majority of these patients underwent a cystectomy (91%, 20/22), while only two patients, with dermoid cysts larger than 10 cm, underwent an oophorectomy. The above characteristics of the dermoid cysts in this group indicate a high risk of ovarian reserve reduction following a surgical excision [23].

None of the patients included had a malignant ovarian finding. The malignant transformation of ovarian dermoid cysts is rare, with a reported prevalence of 0.17 to 3%, although because of the non-specific radiologic findings, it is difficult to diagnose these cases prior to the surgery [24]. The vast majority of the ovarian tumors arising from ovarian dermoid cysts have been reported to be squamous cell carcinoma, and the risk factors for malignant transformations are postmenopausal status, elevated CA-125 levels, and large tumor masses [24]. In our study population, it should be underlined that endometriosis is also a risk factor for ovarian cancer, as it is associated with an increased risk for ovarian cancer and more specifically for the histological subtypes of ovarian clear cell carcinoma and endometrioid ovarian cancer, as different studies have shown [25].

Among the group of women with both endometriosis and ovarian dermoid cysts, 14 out of 22 (63.6%) expressed a desire for future pregnancy. After evaluating the EFI score for these patients, we found that most of the included patients had a score of 7–8 (11/14, 78.5%). According to Ferrier et al., who examined the cost-effectiveness between the different fertility strategies depending on the EFI score, Assisted Reproductive Techniques (ART) may be a good option, whether immediately after surgery or after a year of attempted natural conception, for patients with an intermediate EFI score (4–8). The live birth rate for women with an EFI score of 7–8 after one year of attempted natural conception was 21.1% (41/194), while after one cycle of ART, it was 37.3% (22/59), and after four cycles, it was 54.2% (32/59). The logistic regression found that prognostic factors that could orient patients to an immediate IVF-ICSI were the incomplete endometriosis resection during the surgery and the low antral follicular count. More than three IVF-ICSI cycles seemed to be cost-ineffective, while a delayed IVF-ICSI was more effective than continuing natural conception attempts [26].

A previous study demonstrated a significantly greater reduction in the Anti-Muller hormone (AMH) levels among women with both ovarian dermoid cysts and endometriosis in comparison to women with only dermoid cysts after undergoing cystectomy [17]. The main principle in the surgical treatment of both dermoid ovarian cysts and ovarian endometriomas in women of reproductive age is that the treatment should be effective in order to reduce the risk of cyst recurrence. and simultaneously, the surgeon has to treat the finding carefully in order to minimize the possible reduction of ovarian reserves. Previous studies have indicated that the recurrence rate for ovarian dermoid cysts varies, ranging from 7% after two years of follow-up to 14% after six years [27]. The surgical management of ovarian endometriomas, including a cystectomy, ablative methods, sclerotherapy with ethanol, and combined techniques, remains a subject of debate, as the ovarian endometrioma per se can reduce the ovarian reserves and the surgery can potentially exacerbate this reduction [28]. Muzii et al. found, after a histological examination of excised samples of ovarian endometriomas and other benign ovarian cysts, that in 54% of women with ovarian endometrioma and in 17% of women with ovarian dermoid cysts, healthy ovarian tissue was excised along with the finding, while in other benign cysts such as serous and mucinous cystadenomas, no healthy ovarian tissue was detected [29]. The same study also found that the level of a surgeon’s expertise is associated with the amount of healthy tissue removed after the excision of endometriomas [30]. In addition, the high recurrence rate of ovarian endometriomas has been taken into consideration, which has been reported to be as high as 21.5% after 2 years and 40–50% 5 years after surgical management [31].

The fact that the majority of women with both of the aforementioned conditions have a desire for future pregnancy, combined with the fact that a surgical excision and the existence of these conditions can potentially compromise ovarian reserves, emphasizes the importance of a preoperative evaluation of the ovarian reserves and comprehensive counseling about fertility preservation options.

### Clinical Implications in the Context of Fertility Preservation

Fertility preservation is initially employed for women with malignancies undergoing gonadotoxic chemotherapy and/or radiotherapy [32]. Many common gynecological entities, such as endometriosis and ovarian cysts, are associated with a reduction of the ovarian reserves. In these instances, fertility preservation serves as a proactive strategy, functioning as a backup in case of natural conception failure and if ART is required in the future.

The first case of oocyte cryopreservation for fertility preservation in women with endometriosis was reported in 2009 [33]. Although clinical data on oocyte cryopreservation in this population are limited, the existing evidence suggests the feasibility of this technique for women with endometrioma and/or deep endometriosis [34,35]. It should be noted that a history of previous surgery and the presence of bilateral endometriomas appear to decrease the response to control ovarian stimulation [34,35]. The optimal protocol for controlled ovarian stimulation in women with endometriosis remains unclear according to the latest guidelines from the European Society of Human Reproduction and Embryology (ESHRE) [36]. The previously recommended ultralong GnRH agonist protocol is no longer recommended due to its uncertain benefit. Pelvic inflammatory disease has been described after vaginal oocyte retrieval in women with the presence of ovarian endometrioma, although such events seem to be rare [37]. The use of antibiotic prophylaxis during vaginal oocyte retrieval for women with ovarian endometriomas is a subject of debate, as is the choice of the most effective antibiotic [34].

Regarding controlled ovarian stimulation in women with dermoid cysts, a retrospective study that compared infertile women with and without ultrasound-diagnosed ovarian dermoid cysts undergoing IVF found no significant difference in the number of retrieved oocytes and oocytes in metaphase II [38]. Another retrospective study comparing women with and without a surgical excision of ovarian dermoid cysts found similar IVF outcomes between the two groups [39]. While previous reports have indicated complications such as a spillage of cyst contents during vaginal oocyte retrieval and chemical peritonitis in women with ovarian dermoid cysts [40], this appears to be a rare occurrence as no cases of this complication were reported in the aforementioned studies.

Ovarian tissue cryopreservation is an alternative method of fertility preservation without ovarian stimulation. The first published case of ovarian tissue cryopreservation in a woman with a 9cm ovarian endometrioma dates back to 2005 [41]. However, this method remains experimental due to the lack of clear evidence regarding the quality of ovarian follicles and the reproductive potential it offers for women with endometriosis [42]. To the best of our knowledge, no cases of women with mature teratoma and ovarian tissue cryopreservation have been published yet.

The ESHRE endometriosis guidelines 2022 underline that the benefit of fertility preservation in women with endometriosis remains uncertain due to limited data on cost-effectiveness and reproductive outcomes [36]. Clinicians should offer the fertility preservation options to younger women with bilateral ovarian findings or a history of ovarian surgery accompanied by a contralateral finding and/or low ovarian reserves [43]. Patients should be made aware that low ovarian reserves may necessitate repetitive ovarian stimulations and that the required number of oocytes increases with a woman’s age [44]. For a realistic chance of achieving a live birth, it is recommended that women under 38 years old consider cryopreserving between 15 and 20 oocytes, while women over 38 years old should aim for cryopreserving between 25 and 30 oocytes [44].

To the best of our knowledge, this is the first study that investigates bidirectionally the co-existence between endometriosis and ovarian dermoid cysts. The histological diagnosis of both endometriosis and ovarian dermoid cysts is a strength of our study. Several limitations of our study should be noted. Firstly, the retrospective design of our study represents a main limitation. Furthermore, our study exclusively enrolled women from one tertiary hospital who underwent laparoscopic surgery, thereby excluding asymptomatic women or those with small non-detectable ovarian dermoid cysts.

Further investigation at the epidemiological level, through large prospective register studies, is needed to draw safe conclusions about the association of both entities. Additionally studies examining the potential molecular and genetic connections between endometriosis and dermoid cysts are essential for understanding their common pathophysiological mechanisms.

## 5. Conclusions

In conclusion, our study found that 26.5% of women with ovarian dermoid cysts also had endometriosis, a notably higher prevalence than observed in the general population. The prevalence of dermoid cysts among women with endometriosis was found to be 2.2%. Clinicians should be aware of this co-existence, particularly when managing patients with a desire for future pregnancy. Preoperative counseling should be an integral part of the care plan for affected individuals, where the potential risk of diminished ovarian reserves should be discussed in detail. Additionally, patients should be informed about the available fertility preservation options prior to undergoing a surgical intervention.

## Figures and Tables

**Table 1:** Baseline characteristics of women with co-existence of endometriosis and ovarian dermoid cysts.

	n = 22
Age (years) *	31.2 (8.8)
BMI (kg/m^2^) *	22.9 (4.45)
Dermoid cysts size (cm) *	4.96 (2.94)
Preoperative hormonal treatment	8/22 (36.3%)
Parity	
- Nullipara	15/22 (68%)
- Primipara	3/22 (14%)
- Multipara	4/22 (18%)
Desire for future pregnancy	14/22 (63.6%)
- Primary infertility	2/14 (14.2%)
- Secondary infertility	1/14 (7.1%)
Dysmenorrhea	11/22 (50%)
Dyspareunia	6/22 (27.2%)
Dysuria	2/22 (9%)
Dyschezia	1/22 (4.5%)

* Data shown as mean (SD).

**Table 2:** #Enzian score of patients with endometriosis and ovarian dermoid cysts.

#Enzian	
- P	
○ 1	9/22 (40.9%)
○ 2	8/22 (36.35)
○ 3	0/22 (0%)
- O	
○ 1	1/22 (4.5%)
○ 2	1/22 (4.5%)
○ 3	0/22 (0%)
- T	
○ 1	2/22 (9%)
○ 2	0/22 (0%)
○ 3	0/22 (0%)
- A	
○ 1	0/22 (0%)
○ 2	2/22 (9%)
○ 3	0/22 (0%)
- B	
○ 1	0/22 (0%)
○ 2	5/22 (22.7%)
○ 3	3/22 (13.6%)
- C	
○ 1	1/22 (4.5%)
○ 2	2/22 (9%)
○ 3	0/22 (0%)
- FA	3/22 (13.6%)
- FB	0/22 (0%)
- FI	0/22 (0%)
- FU	0/22 (0%)

**Table 3:** EFI score of patients with coexistence of endometriosis and ovarian dermoid cysts and desire for a future pregnancy.

EFI (Endometriosis Fertility Index)	
- 9–10	1 (7.1%)
- 7–8	11 (78.5%)
- 6	1 (7.1%)
- 5	0 (0%)
- 4	1 (7.1%)

## Data Availability

The data presented in this study are available on request from the corresponding author. The data are not publicly available according to the requirements of the ethics committee.
